# Clinical value of serum iPTH, ALP and serum markers levels in patients with secondary hyperparathyroidism receiving paricalcitol combined with cinacalcet

**DOI:** 10.5937/jomb0-55510

**Published:** 2025-07-04

**Authors:** Song Ruifang, Chen Fadong, Xue Zengqi, Lu Xiaozhen

**Affiliations:** 1 The Third Affiliated Hospital of Wenzhou Medical University, Department of Nephrology, Ruian, China

**Keywords:** paricalcitol, cinacalcet, maintenance hemodialysis, chronic kidney disease, hyperparathyroidism, parikalcitol, cinakalcet, održavajuća hemodijaliza, hronična bubrežna bolest, hiperparatireoidizam

## Abstract

**Background:**

This study aimed to evaluate the effect of combining paricalcitol with cinacalcet on the levels of intact parathyroid hormone (iPTH) and alkaline phosphatase (ALP) in patients with secondary hyperparathyroidism undergoing maintenance hemodialysis (MHD).

**Methods:**

A retrospective analysis was conducted on the clinical records of 129 patients diagnosed with chronic kidney disease (CKD) and secondary hyperparathyroidism who received MHD between June and December 2022. The patients were categorised into three groups based on their treatment regimen: Group A (paricalcitol alone, n=43), Group B (cinacalcet alone, n=43), and Group C (combined paricalcitol and cinacalcet, n=43). Hemoglobin (Hb), serum albumin (Alb), calcium (Ca), phosphorus (P), iPTH, ALP, serum creatinine (Scr), and blood urea nitrogen (BUN) levels were measured at admission, 1 day, 3 months, and 6 months to compare the outcomes across the three groups.

**Results:**

No significant differences among the groups were observed in Hb and Alb levels at 1 day post-admission (P>0.05). However, after 3 and 6 months of treatment, Hb and Alb levels increased in all groups, with Group C showing the greatest improvement (P<0.05). iPTH, Ca, and P levels were similar across all groups on day 1 (P>0.05), but by 3 and 6 months, all groups showed reductions, with Group C having the lowest levels (P<0.05). Similarly, ALP, Scr, and BUN levels decreased in all groups over time, with Group C again demonstrating the greatest reduction (P<0.05).

**Conclusions:**

The combination of paricalcitol and cinacalcet was effective in reducing iPTH, calcium, phosphorus, and ALP and improving Hb and Alb levels in patients with secondary hyperparathyroidism on maintenance hemodialysis. This treatment approach offers significant benefits in managing SHPT.

## Abstract (sr)

**Uvod:**

Ova studija je imala za cilj da proceni efekat kombinacije parikalcitola i cinakalceta na nivoe intaktnog paratiroidnog hormona (iPTH) i alkalne fosfataze (ALP) kod pacijenata sa sekundarnim hiperparatireoidizmom koji su na održavajućoj hemodijalizi (MHD).

**Metode:**

Retrospektivna analiza sprovedena je na kliničkim podacima 129 pacijenata sa dijagnozom hronične bubrežne bolesti (CKD) i sekundarnog hiperparatireoidizma koji su primali MHD između juna i decembra 2022. godine. Pacijenti su podeljeni u tri grupe na osnovu režima lečenja: Grupa A (samo parikalcitol, n=43), Grupa B (samo cinakalcet, n=43) i Grupa C (kombinacija parikalcitola i cinakalceta, n=43). Nivo hemoglobina (Hb), serumskog albumina (Alb), kalcijuma (Ca), fosfora (P), iPTH, ALP, serumskog kreatinina (Scr) i azotne materije krvi (BUN) mereni su pri prijemu, nakon 1 dana, 3 meseca i 6 meseci kako bi se uporedili ishodi između tri grupe.

**Rezultati:**

Nije bilo značajnih razlika među grupama u nivoima Hb i Alb 1 dan nakon prijema (P>0,05). Međutim, nakon 3 i 6 meseci lečenja, nivoi Hb i Alb povećali su se u svim grupama, pri čemu je Grupa C pokazala najveće poboljšanje (P<0,05). Nivoi iPTH, Ca i P bili su slični u svim grupama prvog dana (P>0,05), ali su se do 3. i 6. meseca u svim grupama smanjili, s tim da je Grupa C imala najniže nivoe (P<0,05). Slično tome, nivoi ALP, Scr i BUN su se tokom vremena smanjivali u svim grupama, pri čemu je Grupa C ponovo pokazala najveće smanjenje (P<0,05).

**Zaključak:**

Kombinacija parikalcitola i cinakalceta bila je efikasna u sni'avanju nivoa iPTH, kalcijuma, fosfora i ALP, kao i u poboljšanju nivoa Hb i Alb kod pacijenata sa sekundarnim hiperparatireoidizmom na održavajućoj hemodijalizi. Ovaj terapijski pristup nudi značajne koristi u upravljanju SHPT-om.

## Introduction

Chronic kidney disease (CKD) has emerged as a significant global health challenge, with its prevalence affecting approximately 11%–17% of the population worldwide. In China alone, an estimated 120 million adults suffer from CKD, with a prevalence rate of about 10.8% among individuals aged 18 and older [1] [2]. This disease is often characterised by low awareness, high medical costs, and poor prognosis, which poses a substantial burden on both patients and healthcare systems [3]. A common complication of CKD is secondary hyperparathyroidism (SHPT), a condition typically marked by elevated intact parathyroid hormone (iPTH) levels and associated symptoms such as bone pain, skeletal deformities, skin itching, and disturbances in calcium and phosphorus balance [4]. SHPT significantly increases the risk of cardiovascular disease and adversely impacts patients’ long-term health outcomes and quality of life [5].

The primary treatments for SHPT include drug therapy, surgery, and interventional methods, with drug therapy being the most commonly used, particularly in patients with advanced CKD who may not be candidates for surgery due to age or physical limitations [6]. Among pharmacologic therapies, vitamin D receptor agonists and calcimimetics, such as cinacalcet, are frequently utilised to manage SHPT [7]. The pathogenesis of SHPT is closely tied to the dysfunction of calcium-sensing receptors (CaSR) in the parathyroid glands, which regulate parathyroid hormone secretion. Dysfunction in CaSR signalling has been shown to contribute to parathyroid hyperplasia and progression of SHPT [8] [9] [10].

Paricalcitol, a third-generation selective vitamin D receptor activator, has shown promise in treating SHPT, reducing iPTH levels and improving bone metabolism in hemodialysis patients. While it effectively controls SHPT, paricalcitol’s capacity to enhance intestinal calcium absorption is limited, and prolonged use can lead to hypercalcemia and hyperphosphatemia, increasing the risk of vascular calcification [11] [12] [13] [14] [15] [16] [17]. Additionally, for patients with severe SHPT and parathyroid nodular hyperplasia, paricalcitol may not provide adequate relief, necessitating parathyroidectomy as a treatment option [18] [19].

In recent years, the introduction of cinacalcet hydrochloride, a novel calcimimetic agent, has provided an alternative therapeutic approach by activating CaSR and inhibiting PTH secretion, leading to improved regulation of calcium, phosphorus, and iPTH levels [20] [21]. Cinacalcet has shown efficacy in managing SHPT, particularly in patients who are unresponsive to traditional therapies like paricalcitol. However, some patients experience poor responses or discontinue therapy due to side effects. As a result, there is increasing interest in exploring the combination of paricalcitol and cinacalcet as a potential treatment strategy for more effective management of SHPT in hemodialysis patients [22].

The main aim of this study is to evaluate the efficacy and safety of combining paricalcitol with cinacalcet in treating secondary hyperparathyroidism among maintenance hemodialysis patients. This study is novel in its approach to investigating the synergistic effects of these two therapeutic agents, which may offer a more comprehensive and effective solution to managing SHPT. By assessing the combined impact on iPTH, calcium, and phosphorus levels and evaluating the potential for reducing side effects and improving long-term patient adherence, this study aims to provide valuable clinical insights and establish a theoretical basis for enhanced management of SHPT in dialysis patients.

## Materials and methods

### General data

The case data of 129 patients with CKD secondary hyperparathyroidism who underwent MHD treatment in the hemodialysis room from June 2022 to December 2022 were retrospectively analysed.

**Figure 1:**
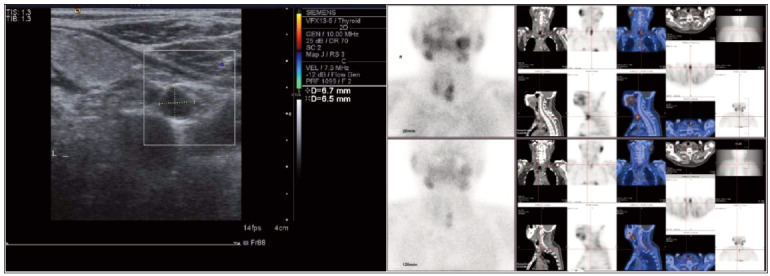
B-scan ultrasonography, ETC examination of secondary hyperparathyroidism.<br>Note: The results of B-scan ultrasonography on the left side suggested hypoechoic inferior lateral pole of the left thyroid; the ETC examination findings on the right side indicated a delayed uptake of MIBI lesion in the lower portion of the left lobe thyroid, prompting consideration of the parathyroid gland’s location and the potential for adenomatous hyperplasia; small nodules posterior to the middle of the right lobe thyroid.

**Diagnostic criteria:** a. Meet the diagnostic criteria for secondary hyperparathyroidism [23]; b. B-scan ultrasonography, imaging and other relevant auxiliary examinations further confirm secondary hyperparathyroidism, as shown in Figure 1(A, B);

**Inclusion criteria:** a. Patients who meet the above diagnostic criteria; b. Patients with complete clinical data can provide complete records of examination and the whole course of disease during hospitalisation; c. iPTH>600 pg/mL; d. Regular hemodialysis > 6 months.

**Exclusion criteria:** a. Patients with cardiovascular diseases; b. Patients with drug contraindications; c. Patients with systemic malignancies; d. Patients presenting with severe hypocalcemia (<1.9 mmol/L), hypercalcemia (>2.7 mmol/L), and hyperphosphatemia (>2.9 mmol/L) were included in the study.

According to treatment methods, patients were categorised into group A (43 treated with paricalcitol), group B (43 treated with cinacalcet), and group C (43 treated with both paricalcitol and cinacalcet). Baseline data across the three groups are presented in Table 1 for comparison.

**Table 1:** The baseline data for the three groups (x̄±s/n).

Group	Number<br>of cases	Gender (cases)		Age (years)	Protopathy			Age on dialysis<br>(years)
		Male	Female		Diabet	Hypertensive	Chronic	
Group A	43	19	24	64.93±9.59	12	22	9	8.65±2.82
Group B	43	21	22	64.30±7.36	14	16	13	8.98±2.85
Group C	43	18	25	67.91±8.93	9	24	10	8.47±2.41
*F/x^2^*		0.439			3.576			
*P*		0.803			0.467			

Note: The results of B-scan ultrasonography on the left side suggested hypoechoic inferior lateral pole of the left thyroid; the ETC examination findings on the right side indicated a delayed uptake of MIBI lesion in the lower portion of the left lobe thyroid, prompting consideration of the parathyroid gland’s location and the potential for adenomatous hyperplasia; small nodules posterior to the middle of the right lobe thyroid.

### Methods

All patients were on a low-phosphorus diet. If hyperphosphatemia occurred at the beginning or during treatment, lanthanum carbonate was given with meals to avoid the effect on blood Ca. Hemodialysis was performed thrice weekly, with each session spanning 4 hours. A dialysate flow rate of 500 mL/min and blood flow ranging from 250 to 300 mL/min were employed. Additionally, the calcium concentration was set at 1.50 mmol/L.

Group A: Treatment with paricalcitol injection (manufactured by Jiangsu Hengrui PharmaceuticalCo., Ltd., approval No. H20183043), 5 mg of paricalcitol injection each time, 2–3 times a week, administered through dialysis catheter 30 min before the end of hemodialysis; blood Ca, P and iPTH were monitored every 4 weeks, and the dose was adjusted according to the laboratory results.

Group B: Cinacalcet (manufactured by Jiangsu Jiayi Pharmaceutical Co., Ltd., Approval No. H20203165) 25 mg/d swallowed; the dose was adjusted according to blood Ca and iPTH results, with a maximum dose of 50 mg/d.

Group C: Treatment of Paricalcitol Injection (manufactured by Jiangsu Hengrui Pharmaceutical Co., Ltd., Approval No. H20183043): 5 mg paricalcitol injection each time, 2~3 times a week, administered in the dialysis tube 30 min before the end of dialysis, plus cinacalcet (manufactured by Jiangsu Jiayi Pharmaceutical Co., Ltd., Approval No. H20203165) 25 mg/d for swallowing, and adjust the dose according to blood Ca and iPTH results. The maximum dose is 50 mg/d.

### Monitoring indicators

The Hb, Alb, Ca, P, iPTH, ALP, Scr, and BUN levels were assessed at 1 day, 3 months, and 6 monthspost-admission. Each patient provided a 5 mL blood sample, which was then centrifuged at 3000 r/min for 5 minutes. Subsequently, the serum was extracted and stored at -20°C for testing. Chemiluminescence assay was conducted using equipment from Shandong Aikeda Biotechnology Co., Ltd. (model: AC-1000), while the remaining parameters were analysed via an automatic biochemical analyser (model: TBA-120FR) from Canon Medical Systems (China) Inc. All procedures adhered strictly to the reagent instructions.

### Statistical methods

EpiData software was used to establish a database, and two persons were assigned for parallelentry to ensure the accuracy of data input. The data gathered for the study underwent statistical analysis through SPSS 26.0 software. Measurement data were expressed as mean ± standard deviation (x̄±s), with intergroup comparisons performed using the sample t-test. Within-group comparisons across various time points were conducted using repeated measures to analyse variance. Enumeration data were presented as frequency or percentage and analysed using the chi-square test, with statistical significance set at *P*<0.05.

## Results

### Demographic and baseline data

The study included a total of 129 patients diagnosed with chronic kidney disease (CKD) secondary hyperparathyroidism who underwent maintenance hemodialysis (MHD) treatment between June 2022 and December 2022. Patients were divided into three groups based on the treatment method:

- Group A: 43 patients treated with paricalcitol.
- Group B: 43 patients treated with cinacalcet.
- Group C: 43 patients treated with both paricalcitol and cinacalcet.

The baseline data for the groups were as follows:

In Group A, there were 43 patients (19 male, 24 female), with an average age of 64.93±9.59 years. The distribution of underlying diseases was 12 with diabetic nephropathy, 22 with hypertensive nephropathy, and 9 with chronic glomerulonephritis. The average age on dialysis was 8.65±2.82 years.

In Group B, there were 43 patients (21 male, 22 female), with an average age of 64.30±7.36 years. The distribution of underlying diseases was 14 with diabetic nephropathy, 16 with hypertensive nephropathy, and 13 with chronic glomerulonephritis. The average age on dialysis was 8.98±2.85 years.

In Group C, there were 43 patients (18 male, 25 female), with an average age of 67.91±8.93 years. The distribution of underlying diseases was 9 with diabetic nephropathy, 24 with hypertensive nephropathy, and 10 with chronic glomerulonephritis. The average age on dialysis was 8.47±2.41 years.

### Comparison of Hb and Alb levels in the 3 groups

**Figure 2:**
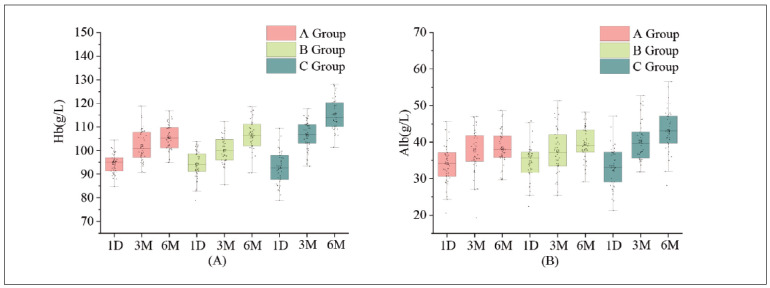
Box Plot of Hb and Alb in the 3 groups at each time point.<br>Please note that (A) represents the box plot illustrating the haemoglobin (Hb) levels across the three groups at each time point, while (B) depicts the box plot displaying the serum albumin (Alb) levels across the three groups at each time point.

A comparison of Hb and Alb levels in the 3 groups is shown in Figure 2 (A, B). Analysis of haemoglobin (Hb) and serum albumin (Alb) levels at 1 day post-admission across the three groups revealed no significant differences (*P*>0.05). However, following 3 and 6 months of treatment, Hb and Alb levels increased in all groups compared to levels at 1 day post-admission, with group C exhibiting higher levels than groups A and B (*P*<0.05). Results from repeated analysis of variance indicated significant differences in *F*time, *F*groups and *F* interaction (*P*<0.05), as outlined in Table 2.

**Table 2:** Hemoglobin (Hb) and serum albumin (Alb) levels among the three groups (x̄±s).

Group	Number<br>of cases	Hb (g/L)			*F*	*P*
		1 day after admission	3 months	6 months		
Group A	43	94.54±4.12	102.06±6.70	105.58±5.29	Fgroup=24.964	Pgroup<0.001
Group B	43	94.20±5.71	100.14±5.51	106.37±6.33	Ftime=204.344	Ptime<0.001
Group	43	92.87±6.98	106.56±5.93	115.40±7.06	Finteraction=12.350	Pinteraction<0.001
Group	Number<br>of cases	Alb(g/L)			*F*	*P*
		1 day after admission	3 months	6 months		
Group A	43	34.09±5.40	37.62±5.86	38.37±4.74	Fgroup=5.598	Pgroup=0.005
Group B	43	34.50±4.83	37.53±6.33	39.53±4.51	Ftime=61.382	Ptime<0.001
Group C	43	33.40±5.48	40.26±5.44	43.14±5.81	Finteraction=4.467	Pinteraction=0.002

Please note that (A) represents the box plot illustrating the haemoglobin (Hb) levels across the three groups at each time point, while (B) depicts the box plot displaying the serum albumin (Alb) levels across the three groups at each time point.

### Comparison of Ca, P and iPTH levels among the three groups

**Figure 3:**
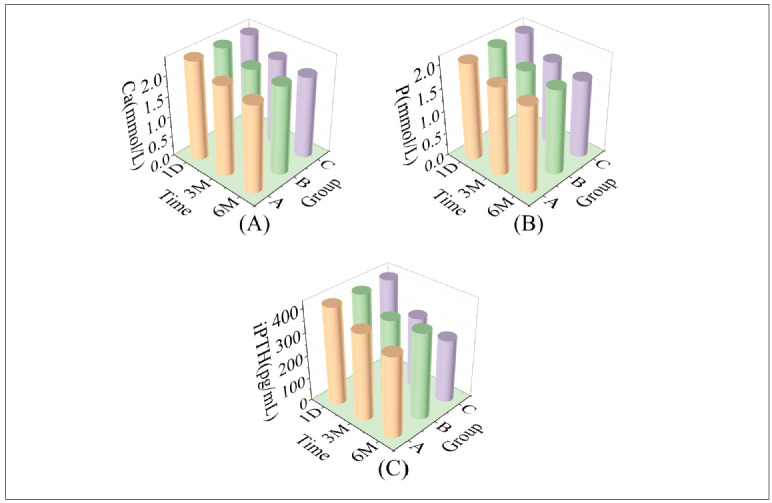
3D bar charts of Ca, P and iPTH in the three groups at each time point.

Figure 3 (A, B) compares the three groups’ Ca, P, and iPTH levels. Analysis of these levels at 1 day post-admission indicated no significant differences (*P*>0.05). However, following 3 and 6 months of treatment, Ca, P, and iPTH levels decreased in all groups compared to 1 day post-admission, with group C demonstrating lower levels (*P*<0.05). Results from repeated analysis of variance revealed significant differences in *F*time, *F* group and *F* interacting (*P*<0.05), as detailed in Table 3.

**Table 3:** The Ca, P, and iPTH levels across the three groups (x̄±s).

Group	Number<br>of cases	Ca (mmol/L)			*F*	*P*
		1 day after admission	3 months	6 months		
Group A	43	2.42±0.23	2.19±0.27	2.11±0.16	*F*group=27.283	*P*group<0.001
Group B	43	2.40±0.18	2.21±0.18	2.15±0.20	*F*time=62.647	*P*time<0.001
Group C	43	2.40±0.17	2.10±0.20	2.00±0.07	*F*interaction=9.300	*P*interaction<0.001
Group	Number<br>of cases	P (mmol/L)			*F*	*P*
		1 day after admission	3 months	6 months		
Group A	43	2.10±0.21	1.92±0.23	1.86±0.17	*F*group=5.332	*P*group=0.006
Group B	43	2.14±0.25	1.93±0.20	1.84±0.21	*F*time=91.528	*P*time<0.001
Group C	43	2.16±0.21	1.81±0.23	1.70±0.18	*F*interaction=3.900	*P*interaction=0.004
Group Number	Number<br>of cases	iPTH (pg/mL)			*F*	*P*
		1 day after admission	3 months	6 months		
Group A	43	420.12±97.73	373.11±93.96	344.36±71.76	*F*group=14.385	*P*group<0.001
Group B	43	415.93±107.08	362.65±88.99	369.79±68.79	*F*time=41.559	*P*time<0.001
Group C	43	420.67±73.75	309.87±64.61	273.33±60.39	*F*interaction=4.644	*P*interaction=0.001

Kindly note the following: (A) exhibits 3D bar plots displaying calcium (Ca) levels at each time point for the three groups; (B) demonstrates 3D bar plots illustrating phosphorus (P) levels at each time point among the three groups; and (C) presents 3D bar plots showing intact parathyroid hormone (iPTH) levels at each time point across the three groups.

### The comparison among the three groups of ALP, Scr, and BUN levels

**Figure 4:**
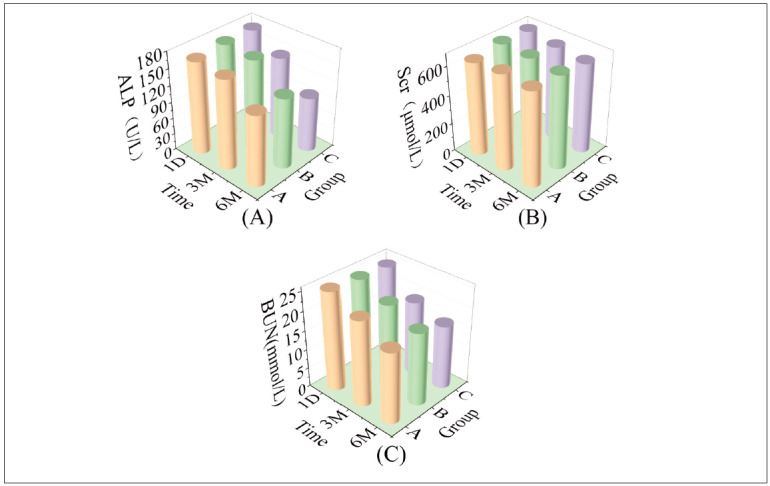
3D bar charts of ALP, Scr and BUN in the three groups at each time point.<br>Note: (A) 3 groups of 3D bar charts for ALP at each time point; (B) 3 groups of 3D bar charts for Scr at each time point; and (C) 3 groups of 3D bar charts for BUN at each time point.

Figure 4 (A–C) illustrates the comparison of alkaline phosphatase (ALP), serum creatinine (Scr), and blood urea nitrogen (BUN) levels across the three groups. Analysing these levels at 1 day post-admission revealed no significant differences (P>0.05). However, following 3 and 6 months of treatment, ALP, Scr, and BUN levels decreased in all groups compared to levels at 1 day post-admission, with group C exhibiting lower levels than groups A and B (*P*<0.05). Results from repeated analysis of variance displayed significant differences in F time, F group, and F interaction (*P*<0.05), as documented in Table 4.

**Table 4:** The ALP, Scr, and BUN levels among the three groups (x̄±s).

Group	Number<br>of cases	ALP (U/L)			*F*	*P*
		1 day after admission	3 months	6 months		
Group A	43	168.37±33.48	162.99±36.13	129.14±26.90	*F*group=9.982	*P*group<0.001
Group B	43	173.76±31.58	169.52±28.97	127.95±24.47	*F*time=114.868	*P*time<0.001
Group C	43	175.97±32.35	147.85±25.35	99.82±27.04	*F*interaction=5.011	*P*interaction<0.001
Group	Number<br>of cases	Scr (mmol/L)			*F*	*P*
		1 day after admission	3 months	6 months		
Group A	43	674.43±131.33	665.43±26.73	654.19±21.34	*F*group=13.772	*P*group<0.001
Group B	43	680.03±132.09	670.52±26.94	650.37±22.15	*F*time=52.154	*P*time<0.001
Group C	43	678.50±127.66	651.00±20.21	627.53±19.17	*F*interaction=4.068	*P*interaction=0.004
Group	Number<br>of cases	BUN (mmol/L)			*F*	*P*
		1 day after admission	3 months	6 months		
Group A	43	25.82±5.10	22.30±3.24	18.48±2.78	*F*group=11.345	*P*group<0.001
Group B	43	25.38±4.88	22.26±3.03	18.89±3.03	*F*time=136.138	*P*time<0.001
Group C	43	25.18±3.76	19.22±3.15	16.58±2.34	*F*interaction=3.219	*P*interaction=0.027

## Discussion

In vivo [24] demonstrated that elevated intact parathyroid hormone (iPTH) levels served as an independent risk factor for relative deficiency or diminished synthesis of erythropoietin (EPO). High levels of iPTH inhibit the activity of Na^+^-K^+^-ATPase and lead to decreased glycolysis and interference with energy metabolism in red blood cells, thus shortening their lifespan [25]. Furthermore, intact parathyroid hormone (iPTH) can disrupt the regular functioning of red blood cells by facilitating the entry of calcium ions into these cells, thereby heightening their osmotic fragility and impeding haemoglobin synthesis. These effects combine to exacerbate red blood cell damage and potential health risks.

This leads to decreased Hb and Alb levels in the body [26]. Showed that 25(OH)D has an important positive regulation effect on renal anaemia, and active vitamin D treatment can increase EPO levels in the body and improve anaemia [27]. Paricalcitol is a selective VD receptor agonist which improves vitamin deficiency and interacts with EPO to promote the synthesis and secretion of EPO, promote erythropoiesis in the bone marrow and increase erythropoietin sensitivity [28]; cinacalcet is a calcamodulin that can significantly improve renal anaemia when treated with cinacalcet. By lowering PTH levels, it promotes an increase in haemoglobin. In addition, cinacalcet can improve renal anaemia and reduce erythropoietin (EPO) resistance [29]. In addition to directly reducing PTH levels, cinacalcet may contribute to erythropoiesis by affecting the migration and maturation of hematopoietic stem cells (HSCs) [30]. Moreover, cinacalcet reduces serum fibroblast growth factor-23 (FGF-23) levels in patients with secondary hyperparathyroidism (SHPT). This action inhibits erythropoiesis, consequently assisting in improving renal anaemia [31]. Demonstrated that after 6 months of paricalcitol and cinacalcet treatment alone, haemoglobin (Hb) and serum albumin (Alb) levels in SHPT patients did not differ from baseline levels (*P*>0.0), whereas in the combination group, there was a significant improvement. Comparison of Hb and Alb levels among the three groups at 1-day post-admission showed no significant differences (*P*>0.0). However, after 3 and 6 months of treatment, Hb and Alb levels in all three groups increased compared to 1-day post-admission, with group C exhibiting higher levels than groups A and B (*P*<0.05). These findings align with previous research, suggesting that the combined therapy enhances Hb and Alb levels.

Reported that in clinical trials for pediatric patients with secondary hyperparathyroidism (SHPT) undergoing hemodialysis [32], 7.4–57.1% of subjects treated with cinacalcet achieved PTH levels within the recommended target range, while 22.2–70.6% experienced a PTH reduction of ≥30%. A retrospective observational study compared 14 children prescribed cinacalcet with 22 children receiving standard care [33]. In children with end-stage kidney disease (ESKD), a decrease in mean serum intact parathyroid hormone (iPTH) levels was observed following 6 months of cinacalcet treatment for secondary hyperparathyroidism (SHPT). The levels dropped from 202 pmol/L (95% confidence interval: 150–253) to 88 pmol/L (95% CI: 41–136), in contrast to the changes noted in the control group (P<0.001). Active vitamin D and its analogues exert their inhibitory effect on the secretion and synthesis of parathyroid hormone by binding to vitamin D receptors in the parathyroid gland. They facilitate adequate absorption of calcium and phosphorus from the intestinal tract, indirectly influencing iPTH secretion and reducing iPTH levels. However, these medications carry the risk of elevating blood calcium levels. Previous studies have shown that paricalcitol, acting as an active vitamin D analogue, possesses unique pharmacological properties by suppressing the NF-kB signalling pathway. This pathway is pivotal in numerous biological processes, such as immune response, inflammation, and apoptosis. Through inhibiting the NF-kB signalling pathway, paricalcitol potentially offers therapeutic benefits in these processes, thereby presenting novel approaches for managing associated conditions.

Protect the functional integrity of glomerular epithelium and accelerate the physiological excretion rate of serum phosphate [34]. However, paricalcitol is a selective VD receptor agonist that binds to intestinal VDRs and increases calcium and phosphorus reabsorption from the gut, inducing hypercalcemia. Cinacalcet is a type II calcium-sensitive receptor agonist in parathyroid cells, which promotes the absorption of calcium by parathyroid glands and the release of intracellular stored calcium by activating CaSR. It opens up cell membrane Ca^+^ channels and extracellular Ca^+^ influx to increase intracellular Ca^+^ concentration and inhibit iPTH secretion. CaSR agonists can also upregulate CaSR gene expression, inhibit parathyroid cell proliferation and promote apoptosis of parathyroid cells. Slows the progression of parathyroid hyperplasia [35]. However, it reduces blood calcium levels significantly and is easy to cause hypocalcemia. Due to the two-way regulation of blood calcium and different drug targets, it has a synergistic effect when used in combination with paricalcitol to stabilise blood calcium levels and reduce the risk of corresponding complications caused by excessively high or low blood calcium. Other researchers observed alterations in the structure of hyperplastic parathyroid glands after 6 months of treatment. Glands ≥ 500 mm^3^ exhibited cystic degeneration attenuation, while smaller glands displayed more significant changes [36]. Ultrasound imaging indicated a transition from hypoechoic to hyperechoic, reduced glandular blood flow, and decreased gland volume, approaching normalcy. Moreover, there was a decrease in Ca, P, and iPTH levels observed in groups A and B (P<0.05). This is consistent with the findings of Chang et al. that paricalcitol, in combination with cinacalcet, achieves a target level of iPTH without increasing the risk of hypercalcemia. It indicates that after the combination of the two treatments, not only the target iPTH level is achieved, but also the dose of vitamin D analogue is reduced, and serum Ca and P levels are lowered, thus reducing the risk of hypercalcemia or hyperphosphatemia [37]. Short-term treatment with paricalcitol combined with cinacalcetincreased serum creatinine and decreased creatinine clearance but did not affect the glomerular filtration rate [38]. The findings of this study revealed that after 3 and 6 months of treatment, the levels of ALP, Scr, and BUN in all three groups decreased compared to levels on the first-day post-admission. Additionally, levels in group C were lower (*P*<0.05), which contrasts with previous research. This difference may be attributed to the indirect enhancement of hemodialysis efficacy by reducing intact parathyroid hormone (iPTH) levels [37].

One limitation of this study is its retrospective design, which may introduce biases in data collection and patient selection, limiting the ability to establish causal relationships. Additionally, the relatively small sample size of 129 patients across three groups may reduce the generalizability of the findings to a broader population. The study also lacks long-term follow-up beyond 6 months, essential to assess the sustained effects and potential risks of combination therapy over extended periods. Furthermore, there may be confounding factors, such as the influence of other medications and comorbidities, that were not fully accounted for, potentially affecting the outcomes. Future studies with larger cohorts and randomised controlled designs are necessary to validate the results and address these limitations.

## Conclusion

This study highlights the effectiveness of combining paricalcitol with cinacalcet in treating secondary hyperparathyroidism (SHPT) in maintenance hemodialysis patients. The combination therapy significantly reduced iPTH, calcium, and phosphorus levels, while improving haemoglobin and serum albumin levels. It provided better control of SHPT and maintained calcium-phosphorus balance, reducing the risks of hypercalcemia and hyperphosphatemia. Although the results are promising, further validation through larger, randomised controlled trials is necessary to confirm these findings and establish long-term safety and efficacy.

## Dodatak

### Funding

The research is supported by Wenzhou City Basic Public Welfare Scientific Research Project (No. Y20220344), Efficacy and safety study of paralcitol combined with sinacasin for the treatment of secondary hyperparathyroidism in patients with maintenance hemodialysis (No. Y20220344).

### Conflict of interest statement

All the authors declare that they have no conflict of interest in this work.
